# Imagery-Mediated Verbal Learning Depends on Vividness–Familiarity Interactions: The Possible Role of Dualistic Resting State Network Activity Interference

**DOI:** 10.3390/brainsci9060143

**Published:** 2019-06-18

**Authors:** Etienne Lefebvre, Amedeo D’Angiulli

**Affiliations:** Neuroscience of Imagination Cognition and Emotion Research (NICER) Lab, Neuroscience Department, Carleton University, Ottawa, ON K1S 5B6, Canada; EtienneLefebvre@cmail.carleton.ca

**Keywords:** DMN, TPN, vividness, familiarity, memory, mental imagery

## Abstract

Using secondary database analysis, we tested whether the (implicit) familiarity of eliciting noun-cues and the (explicit) vividness of corresponding imagery exerted additive or interactive influences on verbal learning, as measured by the probability of incidental noun recall and image latency times (RTs). Noun-cues with incongruent levels of vividness and familiarity (high/low; low/high, respectively) at encoding were subsequently associated at retrieval with the lowest recall probabilities, while noun-cues related with congruent levels (high/high; low/low) were associated with higher recall probabilities. RTs in the high vividness and high familiarity grouping were significantly faster than all other subsets (low/low, low/high, high/low) which did not significantly differ among each other. The findings contradict: (1) associative theories predicting positive monotonic relationships between memory strength and learning; and (2) non-monotonic plasticity hypothesis (NMPH), aiming at generalizing the non-monotonic relationship between a neuron’s excitation level and its synaptic strength to broad neural networks. We propose a dualistic neuropsychological model of memory consolidation that mimics the global activity in two large resting-state networks (RSNs), the default mode network (DMN) and the task-positive-network (TPN). Based on this model, we suggest that incongruence and congruence between vividness and familiarity reflect, respectively, competition and synergy between DMN and TPN activity. We argue that competition or synergy between these RSNs at the time of stimulus encoding disproportionately influences long term semantic memory consolidation in healthy controls. These findings could assist in developing neurophenomenological markers of core memory deficits currently hypothesized to be shared across multiple psychopathological conditions.

## 1. Introduction

Consider the following scenario: You forgot the password for your online banking account. Fortunately, you left yourself a password hint: “childhood telephone number”. You used the number for many years. Yet, even though it felt so familiar, you could recall it only after experiencing a vivid image of yourself dialing it on your old rotary dial phone. This vignette simplifies the vivid incidental recall (VIR) effect. This effect has been replicated in experiments where participants are tasked with generating the visual mental images corresponding to a list of cuing object-nouns, and, after an intervening task or rest delay, they are surprised with the request to recall the words. The cues which correspond to relatively higher subjectively vivid images generally yield a higher probability of being successfully retrieved during unexpected recall [1,2,3,4].

Yet, the neurocognitive processes or variables underpinning effects, such as VIR, have not been addressed directly in terms of underlying memory mechanisms. A possible link can be made with dual process theories of recognition memory, according to which retrieval can take two forms: a recollection of contextual and qualitative details, or a subjective feeling of familiarity about the stimulus in question [5,6]. For stimuli to be recalled during an incidental recall task, the memory of the stimuli must be retrieved with its associated qualitative details, and therefore, must rely on the recollection process of recognition memory. On this account, VIR could occur because more vivid mental imagery strengthens the process of recollection, resulting in higher scores in incidental recall tasks. However, these theories also imply that another key factor should be involved in the vivid incidental recall effect, namely, previous and repeated exposure to the given stimulus, in this case, the familiarity associated with word cuing mental imagery and recall [7]. Still, it is unclear whether a level of familiarity strength similar (or congruent) to that of the vividness in the underlying representation may increase or reduce the success of its recollection, thereby modulating the extent of VIR effects. In other words: Are two different types of relatively weak encodings better than a single relatively strong one for consolidating the same memory? To rephrase in terms of our opening anecdotal vignette: Would we still be able to recall our password hint successfully if we had a vivid image of dialing it, but the number itself lost its familiarity? Would this incongruence between the level of familiarity and vividness hinder recollection? This question is the focus of this paper.

According to associative theories, such as Paivio’s dual coding theory, two memory representations should have cooperative effects on the probability of accurate incidental retrieval [8]. That is, the relative strengths of two memory representations should be additive and should always yield a higher probability of reinstating recollection of the original stimulus during incidental recall. Accordingly, having only one strong encoding should be equivalent to having two different weak ones, and two strong encodings will always be better than two weak ones. Hence, the success of incidental retrieval should increase monotonically with the strength of the memory representation.

Most recently, however, accounts based on the neurobiology of learning and memory challenged associative explanations. In particular, the non-monotonic plasticity hypothesis (NMPH) holds that learning effects (recall accuracy) vary as a non-monotonic function of the amount of excitation associated with competing memory representations [9]. Specifically, NMPH predicts that strong learning effects result from overwhelmingly high levels of excitation in one of the two competing representations; poor learning effects result from moderate excitation in both representations, while no learning effects result from low levels of excitation overall [10]. That is, two strong encodings or a strong single one will always better than two weak ones, but two moderately intense encodings will result in the worst learning outcome. As a result, the neural strength of memory representations should be non-monotonically related to the probability of accurate incidental retrieval.

While the NMPH proposes a theory of memory formation and learning, it is first and foremost a neurobiological theory that is rooted at processes taking place at the level of the neuron. It is still unclear whether theories of synaptic strengthening, such as the non-monotonic plasticity hypothesis, are useful frameworks to utilize when modeling higher order phenomenon, such as memory consolidation and retrieval. Recent work using large functional brain networks have identified clear functional and spatial differential specialization for both memory consolidation and memory retrieval processes. More importantly, clear differences in neural specializations are observed when assessing the influence of the accuracy of memory retrieval (performance) as compared to the cortical activation observed at the time of encoding and retrieval [11,12].

A related, known challenge for theories which attempt to reduce the content of recollections to relatively simple neural mechanisms is that they do not adequately describe the relationships between phenomenal consciousness, for example, in the present context, what seems phenomenally available in imagery (i.e., vivid), and what is ultimately accessed in the underlying memory representations (see [13] for the general argument). This is indicative that building robust and replicable models of memory processes at the level of large functional brain networks can provide insightful analysis that may be able to bridge the gap between the phenomenal, the behavioral, and the neural.

Over the last decade, large functional brain networks have been reliably identified across a variety of independent cognitive states using both functional magnetic resonance imaging (fMRI) and Electroencephalography (EEG). These large functional brain networks, coined resting-state networks (RSNs), have been shown to be accurate biomarkers for different states of consciousness, and more importantly accumulating evidence suggests that abnormal RSN activation patterns underly numerous psychopathologies [14,15]. A growing consensus suggests that functional brain activity can be divided into two anti-correlated RSNs: namely, the default mode network (DMN) and the task-positive-network (TPN) [16]. The DMN is generally referred to as a baseline state or a mind wandering state that involves dynamic activity between multiple cortical and subcortical areas that principally involves the medial temporal subsystems (hippocampus, parahippocampal cortex, and retrosplenial cortex), and the dorsal medial subsystems (dmPFC, TPF, and the temporal pole) [17]. Functionally, the DMN is thought to be responsible for the regulation of emotional processing, self-referential mental activity, and the recollection of prior experiences [18]. The TPN is generally considered to reflect externally mediated cognition that involves activity in a number of subsystems, including the dorsal attention network (intraparietal sulcus, sections of the precentral and frontal sulcus, and middle frontal gyrus), the posterior visual network (retinotopic occipital cortex and the temporal-occipital region), the auditory–phonological network (bilateral superior temporal cortex), and the motor network (regions of the precentral, postcentral, and medial frontal gyri, the primary sensory-motor cortices, and the supplementary motor area) [19].

The literature on the role of RSNs in incidental recall paradigms is sparse. One study has investigated the role of RSNs in the incidental retrieval of episodic memories, concluding that relative deactivation of the DMN results in poor incidental recall scores at the time of retrieval [20]. These results confirm the function of the DMN, namely that suppressing it serves to reduce task-irrelevant processing during sensory intensive tasks. While brain activity during incidental recall appears to be TPN dominant (given the high attentional demand of such a task), little is known about how RSNs influence incidental recall at the time of encoding. According to Craik and Lockhart’s levels of processing effect, accurate incidental recall of semantic memories is strongly influenced by modulatory variables at the time of encoding. For example, the familiarity of a semantic memory will influence the accuracy by which it can be successfully incidentally recalled [5]. Additionally, memories with greater mental imagery vividness also tend to be recalled at a higher percentage as imagery vividness is often tied to emotional salience [21]. Such modulatory variables are thought to influence the dominant RSN activation pattern at the time of memory encoding [22,23].

Despite the generalized anti-correlated nature of the DMN and the TPN, significant variation in task-dependent RSNs have been observed in both experimental conditions involving healthy controls [24,25] and in numerous psychological disorders [26,27,28,29,30,31,32,33,34,35,36]. Task-based RSN variation in healthy controls is thought to represent inherent genetic variability [37], with some individuals demonstrating dominant TPN activation, others demonstrating dominant DMN activation and some reporting mixed RSN activation for a given task.

One such task that elicits large individual variation in dominant RSN activation is visual mental imagery. Very recently, it has been shown that individuals capable of highly vivid imagery visualization capabilities overwhelmingly utilize the TPN, while individuals with low mean vividness ratings overwhelmingly utilize the DMN [38]. Similarly, in memory recognition paradigms the strength associated with stimulus familiarity demonstrates a dichotomous RSN activation pattern. Specifically, individuals with strong familiarity judgments at the time of stimulus presentation demonstrate a dominant TPN activation, and individuals with weak familiarity judgments demonstrate a dominant DMN activation [39]. Research on the relationship between the strength of neuropsychological variables (in this case familiarity and vividness), at the time of stimulus encoding, and their corresponding RSN activation is of critical importance to understand how learning occurs at the level of large-scale neural networks. Our study is among the first to investigate how the strength associated with traditional modulatory psychological variables affects RSN activation and whether differential RSN activation has significant effects on memory consolidation.

In the present study, we report a secondary data analysis on a corpus of cuing-nouns, which examines how the naturally varying strength of word familiarity and imagery vividness influences performance in an incidental free-recall task. We entertain a novel theory as to how VIR can be explained, incorporating aspects of both dual-rote associative models and neurobiologically-inspired NMPH. We propose that VIR is best explained by a dualistic neuropsychological model mimicking the global activity in two large RSNs, the DMN and the TPN. Our proposed model of memory consolidation posits that it is not solely the strength of neuropsychological variables that influences future memory consolidation, it is the dominant pattern of RSNs at the time of encoding that accounts for most of the variability in memory consolidation scores. We hypothesize that dominant RSN activity plays a critical role in memory consolidation given the substantial findings in the clinical literature which demonstrate that abnormal RSN connectivity, particularly high frequencies of DMN and TPN activation, result in memory deficits [24,25,26,27,28,29,30,31,32,33,34]. As a result of these findings, our model hypothesizes that conditions in which stimulus encoding reflects competing activation of DMN and TPN will result in poor memory consolidation scores. That is, two encodings, even if weak, will result in better learning outcomes compared to a mix of one strong and one weak encoding.

**Figure 1:**
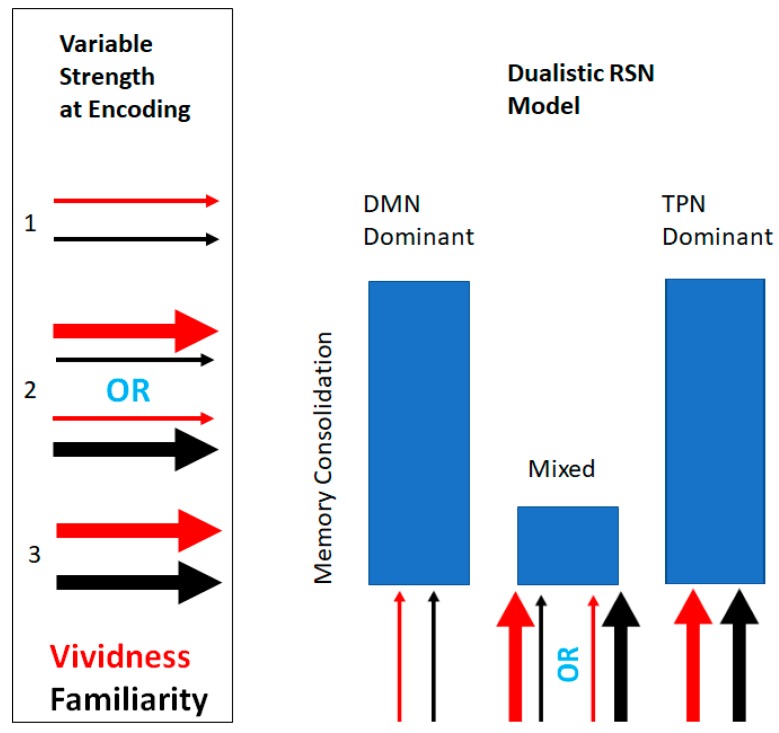
Schematic depicting the dualistic resting-state networks (RSN) neuropsychological model of memory consolidation. The model depicts its predicted memory consolidation score in relation to the three possible combinations of variable strength at the time of encoding. The width of the arrows indicates the strength of the variables at encoding.

The approach of testing our dualistic RSN neuropsychological model of memory consolation by relying on psychological evidence is supported by previous research which has established that some psychological variables of low strength have the ability to selectively engage the DMN, while other psychological variables of high strength have the ability to selectively engage the TPN. Overall, our study aimed to investigate how RSN variability at the time of encoding affects memory consolidation, as measured by scores on an incidental recall task. We expected to find that higher RSN variability/competition, represented by noun-cues associated with mixed scores on our neuropsychological variables (low vividness and high familiarity, or vice versa), would be associated with poor memory recall, whereas congruent levels (high vividness and high familiarity; low vividness and low familiarity) would be associated with higher memory recall (Figure 1).

**Figure A1:**
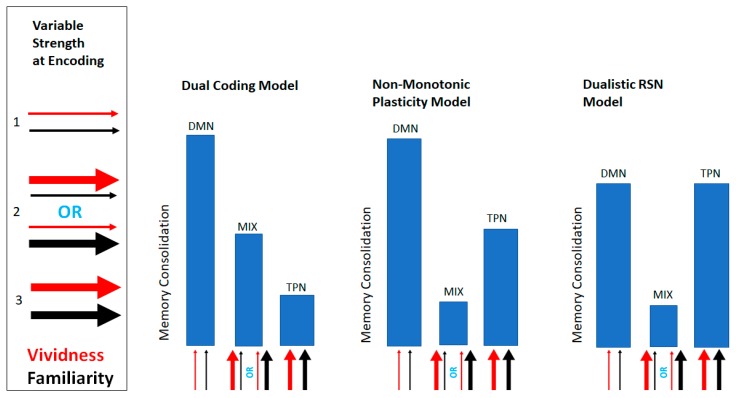
Schematic depicting the three contrasting models of memory consolidation. Each model depicts its predicted memory consolidation scores in relation to the three possible combinations of variable strength at the time of encoding. The width of the arrows signifies the strength of the variables at encoding.

The proposed analysis permits us to contrast our predictions using our dualistic RSN neuropsychological model with those derived from Paivio’s dual coding theory and the NMPH, as they relate to VIR. Since as mentioned, NMPH proposes to explain (i.e., reduce) retrieval in terms of basic neurobiological mechanisms, such a framework, can be extended by incorporating current brain imaging research which indicates that higher mental imagery vividness ratings are associated with an overall increase in neural excitability in the relevant cortical areas [40]. Similarly, familiarity related cortical regions, such as the left angular gyrus, have been shown to increase in neural excitability when participants report that a memory is experienced as more familiar [41]. Thus, relating the above neural excitability patterns with the observed memory performance, if NMPH were to be correct, we would expect higher recall probabilities from noun-cues associated with high vividness and high familiarity ratings (high neural excitability). We would expect low recall probabilities from noun-cues associated with low vividness and high familiarity ratings or vice versa (moderate excitability), and we would expect average recall probabilities from noun-cues associated with low familiarity and low vividness ratings (low neural excitability). In contrast, if Pavio’s dual coding theory were to be correct, we would expect that a higher probability of recall would manifest when both types of memory representations, imagery vividness and imagery familiarity, are strengthened. The recall probability should increase monotonically in relation to the strength of both imagery vividness and imagery familiarity (for comparison of model predictions see Figure A1).

## 2. Method

### 2.1. Design and Analytic Strategy

The present investigation consisted of secondary analyses of a corpus of well-characterized stimuli. The analyses involved a by-item approach followed by a confirmatory linear mixed logistic regression model.

In the by-item analysis, we applied a mixed ANOVA model on the means of recall probability and incidental noun recall and image latency times (RTs) for the stimulus words collapsed across all participants. This procedure, which is ordinarily the most widely used for by-item analysis [42], permits to avoid violating the assumption of independence needed to perform statistical hypothesis testing. By averaging all observations for each stimulus word, it was ensured that only one instance of a participants’ data was used per stimulus word. Thus, the stimuli were the units of analysis, as they were treated as random variables (as if they were “subjects”). The generalizability of results, therefore, referred to both subjects and items populations, washing out individual difference effects (see [42]).

To confirm the ANOVA model, the linear mixed logistic regression approach consisted of analyzing each individual observation nested within participants and stimuli, instead of comparing the averaged responses by stimulus word. This supplementary method was adopted because of its ability to account for within-subjects effects, thereby enabling statistical testing within and between subjects without violating the assumption of independence, further allowing for stronger statistical power than the ANOVA model. (For details on the particular use of linear mixed regression models followed here see [43]).

The database used in this study is available on the archived website [44].

### 2.2. Stimuli

#### 2.2.1. Initial Stimulus Selection

Fifty noun cues were selected from an initial body of 150 noun description-cues from previous extensive research [3,45,46,47,48]. The selection of the nouns for inclusion in the present database was operated through a series of stages. The first stage was one of data reduction to minimize the possibility of confounding attributes. The stimuli in the initial set were saved with an id number and variable columns of attribute variables in an excel file and successively underwent a series of automatic match via the merge command using IBM SPSS Statistics version 25 (Chicago, IL, USA) with regards to noun or compound word frequency, imageability, concreteness, emotional valence (all neutral), and readability attributes all drawn from updated online version of the dictionary file from the Medical Research Council (MRC) Psycholinguistic Database Version 2.0 (MRC2.DCT) [49].

The processed items included single and two-noun descriptions comprising both animate (e.g., dog, cat) and inanimate objects (e.g., car, bottle). To be included in the final database, the stimuli had to be above the mean imageability concreteness and meaningful attributes. All MCR values lie in the scaled range 100 (actual rating of 1) to 700 (actual rating of 7) with the maximum entry of 660 (i.e., 6.6), a mean of 490 (i.e., 4.9) and a standard deviation of 99 (i.e., 0.99). Extensive research in our lab revealed no reliable differences between these two subsets of stimuli in terms of the vividness or latency of elicited imagery. Other secondary analyses indicated that these descriptions are generally rated as relatively emotionally neutral, with negligible inter-item variability along a simple emotional rating scale [50]. These stimuli underwent further automatic merge and selection for several other aspects known to have potentially confounding correlations with other factors in the study. Verbal cues with higher concreteness levels are recalled at significantly higher rates [51,52]. Imageability, which refers to how easily a mental image can be generated from a word, correlates with concreteness [53]. We used the norms reported in the MRC2.CTC to confirm and validate that the selected cuing words had approximately the same scores on these factors [54]. This indirectly controlled for age of acquisition (average age a word enters a subject’s lexicon), as the latter is very strongly predicted by both imageability and concreteness [55,56]. Nonetheless, we still used age of acquisition score norms from the MRC Psycholinguistic database to check for potential confounding effects. All diagnostic analyses showed age of acquisition had no significant or relevant effects. Hence, this variable was dropped from further analysis. At the end of this initial stage, only sixty descriptions were retained.

The second stage involved the collection of direct vividness ratings from raters as part of an imagery and incidental recall experiment using the sixty items which survived the first selection stage. It is important to point out that the MRC2.DCT does not contain vividness norms for the body of nouns contained in it. The procedure and protocol used in the stage are described in detail in the following section.

#### 2.2.2. Vividness Rating Procedure

**Figure 2:**
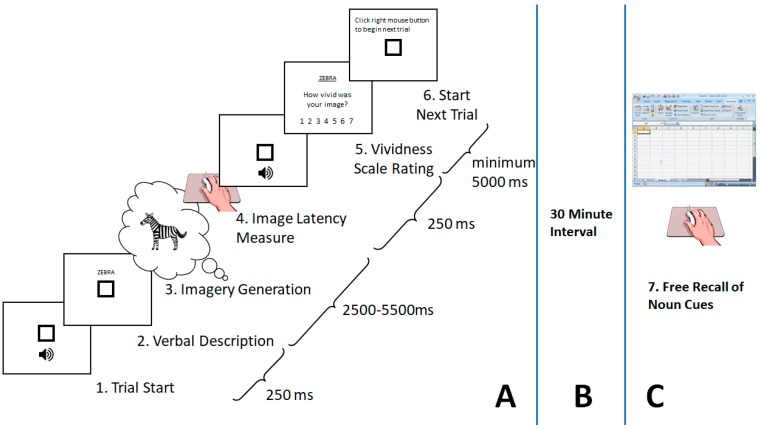
Schematic depicting the overall design of the imagery-generation incidental recall task. (**A**) Participants pressed the right mouse button to begin each trial (1). Upon clicking the mouse, an alerting beep was sounded, followed 250 ms later by the display of a noun-cue at the center of the screen (2). Participants were instructed to read the cue silently and as quickly as possible. They were immediately asked to generate an image that corresponded to the noun-cue (3). When participants felt that their mental image generation was at its most vivid state, they pressed the right mouse button (4). Upon pressing the button, another alerting beep was sounded, followed 250 ms later by a horizontal array of seven choices appearing near the bottom of the screen (5). From left to right, each button was labeled with one of seven vividness level descriptions in a seven-point scale format: ((1), “no image”; (2), “very vague/dim”; (3), “vague/dim”; (4), “not vivid”; (5), “moderately vivid”; (6), “very vivid”; and (7), “perfectly vivid”). Following the vividness response during the rating procedure, the array of buttons disappeared, and the display reverted back to a screen instructing the participant to click the mouse when they were ready to begin the next trial (6). A minimum of 5 s was needed between vividness response and the start of the next trial. (**B**) After completing the image generation phase, participants were told to take a break and fill out paperwork, including a debriefing session. (**C**) Exactly 30 min from their last trial, participants were asked to recall as many of the noun cues as possible on a blank excel spreadsheet (7).

Since vividness was our main independent variable and was not derived from commonly available databases, we here report a summary of the rating procedure used to obtain the corresponding data for the sixty stimuli which survived the first selection stage, which is graphically represented in Figure 2A. This protocol was modeled after the paper and pencil procedures used in the normative studies merged in the MRC2.DCT (additional details of the experimental procedures can be found in [3]). The protocol was approved by the Carleton University Research Ethics Board, in strict adherence with the Tri-Council Policy Statement: Ethical Conduct for Research Involving Humans [57].

##### Sample of Vividness Raters

Participants serving as raters were 26 first-year university students (age range = 17–25; 14 female and 12 male). None had participated in an imagery study before. Participants signed up through a subject pool within 3 weeks of beginning introductory psychology courses, with 2% credit toward their final grade used as an incentive. No significance was found for gender or age against any factors, so these variables were dropped from further consideration. Participants under the age of 18 had to provide a written informed consent letter signed by their legal guardian to participate.

##### Image Generation Phase

Participants were seated facing a computer monitor and pressed the right mouse button to begin each trial. Upon clicking the mouse, an alerting beep was sounded, followed 250 ms later by the display of a noun-cue at the center of the screen. Participants were instructed to read the cue silently and as quickly as possible. They were immediately asked to generate an image that corresponded to the noun-cue. When participants felt that their mental image generation was at its most vivid state, they pressed the right mouse button. Upon pressing the button, another alerting beep was sounded, followed 250 ms later by a horizontal array of seven choices appearing near the bottom of the screen. From left to right, each button was labeled with one of seven vividness level descriptions in a seven-point scale format: ((1), “no image”; (2), “very vague/dim”; (3), “vague/dim”; (4), “not vivid”; (5), “moderately vivid”; (6), “very vivid”; and (7), “perfectly vivid”), as seen in previous research [47,58]. Participants were familiarized with the rating system during pre-test practice sessions. Participants used the mouse to click on one of these seven buttons and were instructed to rate any failure to generate an image as a “no image.” There was no deadline for their response.

##### Stimulus Familiarity Matching and Diagnostic Procedure

The third stage of the selection involved finding a complete archival match of familiarity for the sixty stimuli, again using the MRC2.DCT and using the same merging procedure outlined in Section 2.2.1. to consolidate the present database. MRC2.DCT is an online dictionary file being provided for public research use along with some programs which can be used either to access the dictionary or as examples on which to model programs which match users’ specific needs. The dictionary file does not contain any information which is original to it but was assembled by merging a number of smaller databases published in the psycholinguistic and imagery literature [59]. The original procedure for rating the items consisted of paper and pencil protocol similar to the one we used, albeit in computerized form. In the original norms, the equivalent range of the ratings was 1.00 to 7.00. This database dictionary differs from other machine usable dictionaries in that it includes not only syntactic information but also psychological data for the entries. The file contains 9392 words which possess imagery and other attributes and familiarity ratings except for vividness. The columns 26 to 28 labeled as “FAM” Familiarity stands for ’printed familiarity’. The FAM values were derived from merging three sets of familiarity norms: Paivio, Yuille and Madigan, Toglia and Battig, and Gilhooly and Logie [60,61,62]. The method by which these three sets of norms were merged is described in detail in Appendix 2 of the MRC Psycholinguistic Database User Manual [63]. FAM values lie in the range 100 to 700 with the maximum entry of 657, a mean of 488, and a standard deviation of 99: Note that they are integer values.

The fourth and final stage involved analysis of the distribution of the vividness and familiarity values to avoid range restriction and diagnostics to eliminate outliers. The latter stage narrowed the final number of stimuli in the database further to fifty.

##### Filler Stimuli

In addition, we selected ten other noun-cues from previous earlier research (Paivio, Yuille, and Madigan in 1968) to use as buffer items during the incidental recall phase of the experiment (i.e., to filter out recency and primacy effects during recall) [60]. The sixty cues were presented in random order, preceded by 4 buffer noun-cues and followed by 4 other buffer noun-cues (which were presented in a fixed order).

### 2.3. Dependent Variables

#### 2.3.1. Image Latency Times Measure

A main dependent variable in our study was image latency time. Following the vividness response during the rating procedure, the array of buttons disappeared, and the display reverted back to a screen instructing the participant to click the mouse when they were ready to begin the next trial. In an effort to minimize imagery persistence between trials, stimuli were presented in random order with a minimum inter-trial interval of 5 s, as was done in Craver-Lemley and Reeves [64]. Participants were not informed that latency times (RTs) were covertly measured from when the stimulus was presented to when they gave the first response prompting the appearance of the vividness buttons screen (see (4) in Figure 2). Button presses were justified as the way of communicating to the experimenter a complete image was formed, which was ready to be rated, and prompted the appearance of the vividness scale buttons.

#### 2.3.2. Free Incidental Recall Measure

The main dependent measure in our study was correct recall rates of the noun cues presented during the vividness rating procedure. After completing the image generation phase, participants took a 20 min break. Afterward, they were asked to return to the lab to fill out additional paperwork, to receive course credit, and complete the debriefing process. Prior to the image generation phase, participants had not been informed that they would be required to recall any of the stimuli. Upon their return, precisely 30 min from the end of the image generation phase, they were asked to complete the incidental recall task, wherein they were required to recall and record as many of the previously read noun cues as possible on a blank excel spreadsheet.

Each phase of the previously described procedures was exclusively conducted by one of two paid undergraduate research assistants. Both research assistants received training in their module, yet were unaware of the specific purposes and hypotheses of the study.

## 3. Results

In this section, before reporting the results related to the main hypothesized effects, we describe the characteristics of the by-item analysis in terms of descriptive and correlational statistics.

### 3.1. Stimuli Characteristics: Descriptive and Correlations

For an illustration of the relationship between variables, tables are shown in the following section. Table 1 contains descriptive information of the variables under considerations, including scores of the variables measured. Table 2 shows the correlations between all variables.

### 3.2. Mental Imagery Vividness and Familiarity

The mean vividness score across all noun-cues significantly correlated with each noun-cue’s familiarity rating (*r*_p_ (50) = 0.48, *p* (two-tailed) < 0.001, η_p_^2^ = 0.23), as shown in Table 2. This correlation confirms the relationship between vividness and familiarity posited by previous research, suggesting that our dataset demonstrates the typical relationship observed between the two variables [7].

### 3.3. Mental Imagery Vividness and Reaction Time

The mean vividness score across all noun-cues significantly correlated with each noun cue’s mean reaction time (*r*_p_ (50) = −0.39, *p* (two-tailed) < 0.001, η_p_^2^ = 0.15), as shown in Table 2. This correlation, known as the “vivid-is-fast” phenomenon, has been observed in previous research and therefore helps to validate our experimental dataset [65].

### 3.4. Effects of Resting State Networks on Learning Outcomes 

#### 3.4.1. By-Item Analysis Approach

Following our hypotheses, to assess how RSN activation pattern’s influence mean recall probability across noun-cues, we devised a one-factor ANOVA with three levels, each representing distinct resting state activation patterns. As was described in the introduction, when a dominant DMN activation is observed in human participants, both mental imagery vividness and object familiarity are experienced consciously as a weak stimulus. Inversely, when a dominant TPN activation is observed, both mental imagery and object familiarity are experienced consciously as a strong stimulus. Given this relationship, it is possible to infer how RSNs affect learning outcomes by investigating the relationship between mean familiarity, mean vividness, and mean recall probability ratings. Applying a widely-used continuous variable partitioning technique [66], we created a variable with three levels that aimed to represent the following three distinct resting state network activation patterns: 1. TPN dominant activation (composed of noun cues which have high vividness and high familiarity ratings); 2. Mixed TPN and DMN activation (composed of noun cues with high vividness/low familiarity or low vividness and high familiarity); 3. DMN dominant activation (composed of noun cues with low vividness and low familiarity ratings). The decision to create a three-leveled variable characterized by the strength of mean familiarity and mean vividness scores was influenced by the NMPH model utilized in Norman and Newman [9]. In their model, the moderate/mixed level is of critical importance to assess non-monotonic relations between the strength of a stimulus and subsequent learning outcomes. For this reason, we judged that three levels would be ideal given that this number of levels would adequately contrast differences between states of stimulus competition (high/low) and states of stimulus collaboration. The descriptive information detailing the relationship between the resulting RSN variables and recall probability is shown in Table 3.

**Figure 3:**
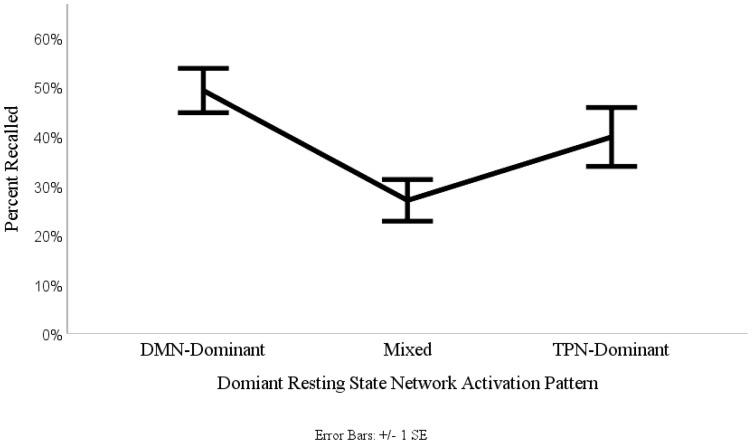
Percentage of words recalled in relation to the dominant resting state network activation pattern.

The percentage of noun-cues recalled for each of the three RSN levels were entered on a 1 (recall probability) × 3 (RSN levels: low, mixed and high activation) one-way ANOVA. This procedure yielded a significant effect of resting state network type on recall probability (*F*(2,47) = 4.51, *p* = 0.016). Additionally, the ANOVA resulted in a large effect size, η_p_^2^ = 0.16 indicating that 16% of the variance in recall probability is attributable to dominant RSN activation. To investigate whether the mixed resting state activation level impaired recall probability, as previously hypothesized (i.e., our switching hypothesis), a polynomial quadratic planned contrast was further applied to the data. The contrast demonstrated a significant quadratic trend (*F*(1,47) = 7.18, *p* = 0.01, η_p_^2^ = 0.13). This quadratic trend can be clearly observed as represented in the continuous connecting line in Figure 3. To investigate how well our results fit within the dualistic RSN neuropsychological model of memory consolidation, we conducted Dunnett’s post-hoc testing using the DMN-Dominant level as our main comparison criteria. Dunnett’s post hoc test revealed that there were no significant differences between the DMN-Dominant and TPN-Dominant levels (Dunnett’s *t*(47) = 1.45, *p* = 0.154), however there was a significant difference between the DMN-Dominant and the Mixed levels (Dunnett’s *t*(47) = 3.01, *p* = 0.004). These results confirm the hypothesized quadratic pattern of the dualistic RSN neuropsychological model of memory consolidation, as shown in the (Figure 3).

To investigate if the quadratic trend was present across different percentiles of the recall probability distribution, the data were converted into proportions as detailed in Table 4. Planned quadratic proportion contrasts were performed on each third of the recall probability distribution. For both the top third (i.e., high recall probability) and middle (i.e., medium recall probability) percentile there were significant positive quadratic trend (*z* = 3.24, *p* < 0.001, η_p_^2^ = 0.21; and *z* = 2.42, *p* = 0.008, η_p_^2^ = 0.12, respectively). Conversely, the low recall group showed a significant negative quadratic trend (*z* = −4.3464, *p* < 0.001, η_p_^2^ = 0.38). To adjust for multiple comparisons, a two-tailed Bonferroni correction was applied to each contrast using 95% confidence intervals. Given that the data being used are proportions, the confidence intervals were treated as the hypothesis test wherein if an interval crossed the 0 threshold it would signify that the null hypothesis failed to be rejected and therefore, the contrast would be interpreted as not significant. The following equation was used to test for multiple comparisons: $\sum p\lambda \pm z_{\frac{\alpha }{2g}\;}\sqrt{s_{p}^{2}\lambda^{2}}$, for further clarification on planned contrast in proportions see Rosenthal and R Rosnow (1985) and Hays (1994) [67,68]. The results of the two-tailed Bonferroni correction are as follows: High recall probability (0.445 ± (−0.301)), Medium recall probability (0.6031 ± 0.2477), Low recall probability (−1.0476 ± 0.2394). Each contrast’s 95% confidence interval did not cross the 0 threshold, indicating that the quadratic contrast amongst each level of the recall variable were significant at *p* < 0.025. The two significant positive quadratic trends in the top 2 percentiles of the recall probability distribution in addition to the significant negative quadratic trend in the bottom percentile demonstrate that noun cues associated with competing RSN activation patterns result in poor recall probability.

#### 3.4.2. Linear Mixed Logistic Regression Approach

To assess how RSN activation patterns influence recall probability across each trial observation (*N* = 1300) we devised a linear mixed logistic regression model where recall of stimuli served as our binary target variable (1 = recalled, 0 = not recalled) and where dominant RSN activation patterns served as our fixed predictor variable. Similar to the by-item analysis, our RSN predictor variable contained three levels, each representing distinct resting state activation patterns. To demarcate each level of the predictor variable, we used the same partitioning technique used in the by-item analysis [66]. Specifically, the underling neuropsychological variable strength used to model the three RSN levels are as follow: 1. TPN dominant activation (composed of trial observations which have high vividness and high familiarity ratings); 2. Mixed TPN and DMN activation (composed of trial observations with high vividness/low familiarity or low vividness and high familiarity ratings); 3. DMN dominant activation (composed of trial observations with low vividness and low familiarity ratings). The descriptive information detailing the relationship between the resulting RSN variables and recall proportions are shown in Table 5.

Controlling for nested within-subjects data, we tested whether recalled noun-cues following a binomial logit link function could be predicted by the fixed effect categorical RSN predictor variable which reflected our three RSN levels (low, mixed, and high activation). This procedure yielded a significant fixed effect of resting state network type on noun-cue recall (*F*(2,1297) = 3.30, *p* = 0.037). To investigate whether these results supported the dualistic RSN neuropsychological model, we conducted Fisher’s least significant difference (LSD) post-hoc testing. The LSD post-hoc test was used over other more conservative statistical tests for two primary reasons. First, since our predictor variable only consisted of three groups, the LSD procedure permits to preserve the experiment-wise type I error rate at nominal levels of significance, which nullifies the use of post-hoc tests that control for the family-wise error rate [69]. Second, for each level of our RSN predictor variable, the variance-covariance matrix demonstrated that covariance scores were all equal, and variance scores did not significantly differ among each other, suggesting that our data did not violate the assumption of compound symmetry needed for appropriate application of the LSD post-hoc test [70]. The LSD post-hoc test revealed that there were no significant differences between the DMN-Dominant and TPN-Dominant levels (LSD’s *t*(1297) = 0.178, *p* = 0.859), however there were significant differences between both DMN-Dominant (LSD’s *t*(1297) = 1.97, *p* = 0.049) and TPN-Dominant (LSD’s *t*(1297) = 2.31, *p* = 0.021) when contrasted with the Mixed RSN level. These results confirm the hypothesized quadratic pattern of the dualistic RSN neuropsychological model of memory consolidation. Additionally, the results generated using the linear mixed logistic regression model helped to validate the findings derived from the by-item approach. Both statistical approaches demonstrated that learning outcomes are significantly predicted by the characteristic non-monotonic quadratic pattern of our dualistic RSN model of memory consolidation.

### 3.5. Effects of Resting State Networks on Mental Imagery Latency (Reaction Time)

The mean reaction times associated with each noun-cue for each of the three resting state network levels were entered in a 1 (reaction time) × 3 (resting state network levels: low, mixed, and high activation) one-way ANOVA. Results showed a significant effect of resting state network type on reaction time (*F*(2,47) = 8.06, *p* = 0.001). Additionally, the ANOVA resulted in a large effect size, η_p_^2^ = 0.26, indicating that 26% of the variance in reaction time is attributable to dominant resting state network activation. Dunnett’s post hoc test revealed that visual mental image generation associated with the high strength level, reflecting TPN-Dominant activity, was significantly faster than visual mental image generation associated with both Mixed (*t*(47) = 3.936, *p* = 0.0003) and DMN-Dominant (*t*(47) = 2.299, *p* = 0.013) RSN activity levels (which did not differ between each other: *t* < 1). Additionally, we found equivalent results when testing whether imagery latency could be predicted by RSN activation, within and between each trial, by using a linear mixed regression model which assumed a normal distribution with an identity link (since this time our target was measured on a continuous scale). These results confirmed that image generation latency is quicker when the relevant imagery is associated with high strength.

## 4. Discussion

In the present study, we explored the degree to which imagery visualization and word familiarity affected learning outcomes in a traditional incidental recall paradigm. In doing so, the current study compared contrasting theoretical predictions regarding the relationship between the strength of neuropsychological variables and learning outcomes. Specifically, we contrasted associative theories that claim that the relative strength of neuropsychological variables should be additive [8], non-monotonic theories that claim that the relative strength of neuropsychological variables should be non-monotonic in relation to learning outcomes [9,10], and RSN theories which predict that learning outcomes should improve when competition between DMN and TPN is reduced [25,34,71].

Our main results demonstrate that recall probability is at its highest when the strength of our neuropsychological variables (noun familiarity/imagery vividness) are in a congruence at the time of stimulus encoding. In other words, when the strength of noun familiarity and imagery vividness are both strong or both weak, recall of the stimulus is at its highest probability. Using our dualistic RSN neuropsychological model, we were able to infer that the strength of our neuropsychological variables was associated with DMN activation when variables were of low strength and associated with TPN activation when variables were of high strength. When considering the inferred pattern of RSN activation, our results show that a competing activation pattern between DMN and TPN at the time of stimulus encoding results in significant impairment of recall probability.

An additional secondary finding was that the mean reaction time to complete the mental imagery task was quickest when the strength of our neuropsychological variables was high. In other words, it was quicker to generate visual mental images from noun cues under conditions where TPN was dominant at the time of encoding. The vivid-is-fast correlation between high image latency speed and high strength of neuropsychological variables has been reported in previous research [48,65,72,73]. However, our findings suggest that in addition to high strength variables, dominant-TPN at the time of encoding is also a key predictor of quick imagery generation latency. These results suggest an intriguing, novel explanation for the vivid-is-fast phenomenon. Namely, that it is not only the strength of neuropsychological variables that predict imagery generation latency but rather the dominant RSN pattern utilized during a mental imagery task. However, given the incongruent results between RSN patterns among our dependent variables (imagery latency was only significantly faster in the TPN grouping, while recall probability was significantly greater in both the DMN and TPN groupings), it is unclear whether image latency is related to the same processes responsible for memory consolidation. This is a research question open for future investigations.

In terms of the findings concerning the recall probability variable, our results do not support associative theories, as high memory consolidation is predicted to be exclusively a result of the additive strength of the preceding memory representations. Therefore, it can be concluded that Paivio’s associative dual coding theory does not accurately model the incidental recall effect when operationalizing the strength of both mental imagery vividness and noun cue familiarity.

While our results did demonstrate a non-monotonic relationship between the strength of memory representations and recall probability, the directionality of our non-monotonic relationship did not align with that of Norman’s non-monotonic-plasticity-hypothesis (NMPH). In the NMPH model, low strength memory representations result in lower memory consolidation scores compared to high strength memory representations. In our results, we found no significant differences between the high and low memory representations strength in terms of recall probabilities, suggesting that the NMPH may not accurately model memory consolidation at levels of reduction greater than single neurons. It is important to note that while our results did not precisely match the type of non-monotonic relationship that characterizes a neuron’s level of excitation and the change in its associated synaptic strength, our results still demonstrated a non-monotonic relationship. This could suggest that there is, at the very least, a partial carryover effect of synaptic neuroplastic principles at the level of large scale distributed neural representations, given that non-monotonic results are rare within memory paradigms. However, the most likely interpretation of our results, that accounts for the largest amount of variability in our data, appears to be related to the variability in RSN dominance at the time of stimulus encoding. Utilizing the current RSN brain imaging literature [36,37] we were able to map the strength of our neuropsychological variables (imagery vividness/stimulus familiarity) with their associated dominant RSNs (DMN or TPN). This permitted the comparison of recall probability scores with the inferred dominant RSN at the time of encoding. Our data clearly suggest that when there is an antagonist relationship between the inferred DMN and TPN activation at the time of stimulus encoding, recall probability, and therefore, memory consolidation is significantly impaired.

Our overall findings help to confirm a hypothesis emerging from the mental health literature suggesting that a possible causal mechanism of memory deficits in neuropsychiatric disorders, such as depression, anxiety, attention-deficit-hyperactivity disorder, autistic spectrum disorder, and schizophrenia, are characterized by abnormalities in RSN activation [24,25,26,27,28,29,30,31,32,33,34]. For example, in schizophrenia, there is a significant increase in DMN activity during attention-demanding tasks, which normally recruits the TPN in healthy controls [74]. The increased DMN activation in schizophrenic patients results in a metabolic competition between the two anti-correlated RSNs, which is hypothesized to explain their stunted performance on such tasks [75]. The strongest evidence of the RSN memory interference hypothesis originates from research on ADHD. In a recent randomized, double-blinded, placebo-controlled clinical trial, researchers explored the RSNs of both ADHD patients and healthy controls while they performed attention-demanding tasks. Their results demonstrated that ADHD patients were unable to suppress the appropriate RSN both during the task and in-between trials. More importantly, ADHD patients were shown to have a significant amount of RSN activation variability during the task. This increased RSN variability, or increased competition between DMN and TPN, is hypothesized by the researchers to be responsible for the reduced scores on the behavioral task compared to controls [25]. Critically, however, these clinical findings provide general evidence that RSN homeostasis is important for efficient memory consolidation, but it is still unclear how abnormal RSN activation arises from such a wide variety of neuropsychiatric disorders. Hopefully, further experiments using healthy controls could help to determine the neurophenomenological marker’s responsible for these core memory deficits.

To our knowledge, this study is the first to investigate how RSN variability might affect memory consolidation in healthy controls by manipulating the strength of neuropsychological variables that influence memory formation. While the results tend to validate the RSN memory deficit hypothesis in the mental health literature, there are still methodological limitations that should be considered prior to generalizing the results of this study. An initial limitation is a result of our reliance on indirect measurements of RSNs. While our dualistic RSN neuropsychological model is inferred from reliable brain imaging research [36,37], it is possible that the interaction between imagery vividness strength and stimulus familiarity at the time of stimulus encoding could reveal RSN activity which differs from our predicted inferences. Further studies using direct RSN imaging classification paradigms will need to be used to more precisely measure changes in dominant RSN activation in the function of both behavioral performance and of the strength of the chosen modulatory variables. A potential modulatory variable that could be used in future studies to help model the role of RSNs in memory consolidation is acute cannabis use. It has recently been demonstrated that cannabis is one of few pharmacological substances that has the effect of acutely increasing DMN connectivity during an attentionally demanding task [76]. Therefore, cannabis, in conjunction with psychological variables, such as mental imagery vividness and familiarly strength, could be used to artificially create an environment where individuals experience high degrees of competition between both the TPN and the DMN. Such experimental conditions could offer insight as to how various neuropsychological variables influence RSNs and therefore, memory consolidation processes.

## 5. Conclusions

In this study, we explored how varying degrees of both stimulus familiarity and mental imagery vividness ratings influenced verbal learning scores, as measured with an incidental recall task. Importantly, using relevant imaging research, we inferred dominant RSN activation patterns based on the reported strength of our neuropsychological variables at the time of stimulus presentation. We contrasted our results amongst three theoretical frameworks, namely: Paivio’s associative theories, Normans’ non-monotonic plasticity theory, and the newly postulated RSN memory interference hypothesis. The findings supported the latter hypothesis given that noun cues with the highest probability of being recalled were associated with noun cues which had the least amount of predicted RSN competition during stimulus presentation. The outcomes of this study, although preliminary, support the idea that a critical component of successful and efficient memory consolidation does not rely on the particular RSN present at the time of memory encoding (DMN or TPN), but rather it relies upon having minimal RSN activation variability between DMN and TPN at the time of memory encoding. This distinction could highlight relevant RSN biomarkers in the diagnosis of the mental health disorders affected by memory deficits. The RSN memory interference hypothesis could also help to illuminate our understanding of the broader neurophysiology of memory processes, complementing and expanding single-neuron neuroplasticity theories.

## Figures and Tables

**Table 1:** The descriptive information of the experimental variables.

Variables	M	SD	Min	Max
Vividness mean	5.26	0.52	3.99	6.12
RTs (ms)	3892.59	524.24	2692.87	5271.39
Familiarity mean	5.60	0.74	3.82	6.84
Recall probability	0.40	0.22	0.08	0.9231

Vividness and familiarity ratings: Min = 1 to Max = 7. RTs = Image latency times in milliseconds.

**Table 2:** Pearson Correlations between the experimental variables.

Variables	Vividness	Reaction Time	Familiarity	Recall Probability
Vividness		−0.39 **	0.48 **	−0.16
Reaction time	−0.39 **		0.04	−0.01
Familiarity	0.48 **	0.04		0.01
Recall Probability	−0.16	−0.01	0.01	

** *p* < 0.001 (2-tailed).

**Table 3:** Descriptive statistics of total noun-cues recalled (in percentages) as a factor of resting state network type.

	Descriptive				
Variables	*N*	M	SE	Min	Max
TPN dominant	18	0.40	0.06	0.08	0.92
Mixed	14	0.27	0.04	0.08	0.58
DMN dominant	18	0.49	0.04	0.15	0.77

**Table 4:** Proportions and (total counts) of noun-cues recalled at three different types of resting state network action pattern.

Variables			DRSNAP		
	Variable Levels	TPN	Mixed	DMN	Total
	High	0.06 (3)	0	0.1 (5)	0.16 (8)
**Recall**	Medium	0.14 (7)	0.04 (2)	0.18 (9)	0.36 (18)
	Low	0.16 (8)	0.24 (12)	0.08 (4)	0.48 (24)
	Total	0.36 (18)	0.28 (14)	0.36 (18)	1.00 (50)

**Table 5:** Descriptive statistics of total noun-cues recalled across all trials (in proportion) as a factor of resting state network type.

	Descriptive				
Variables	*N*	M	SE	95% CI	95% CI
TPN dominant	519	0.36	0.02	0.32	0.40
Mixed	528	0.29	0.02	0.25	0.33
DMN dominant	253	0.36	0.03	0.30	0.42

All values have been adjusted for within-subject effects.
